# Growth Phase-Dependent Proteomes of the Malaysian Isolated *Lactococcus lactis* Dairy Strain M4 Using Label-Free Qualitative Shotgun Proteomics Analysis

**DOI:** 10.1155/2014/642891

**Published:** 2014-03-25

**Authors:** Theresa Wan Chen Yap, Amir Rabu, Farah Diba Abu Bakar, Raha Abdul Rahim, Nor Muhammad Mahadi, Rosli Md. Illias, Abdul Munir Abdul Murad

**Affiliations:** ^1^School of Biosciences and Biotechnology, Faculty of Science and Technology, Universiti Kebangsaan Malaysia, 43600 Bangi, Selangor, Malaysia; ^2^Faculty of Biotechnology and Biomolecular Sciences, Universiti Putra Malaysia (UPM), 43400 Serdang, Selangor, Malaysia; ^3^Malaysia Genome Institute, Jalan Bangi, 43000 Kajang, Selangor, Malaysia; ^4^Department of Bioprocess Engineering, Faculty of Chemical Engineering, Universiti Teknologi Malaysia, 81310 Skudai, Johor, Malaysia

## Abstract

*Lactococcus lactis* is the most studied mesophilic fermentative lactic acid bacterium. It is used extensively in the food industry and plays a pivotal role as a cell factory and also as vaccine delivery platforms. The proteome of the Malaysian isolated *L. lactis* M4 dairy strain, obtained from the milk of locally bred cows, was studied to elucidate the physiological changes occurring between the growth phases of this bacterium. In this study, ultraperformance liquid chromatography nanoflow electrospray ionization tandem mass spectrometry (UPLC- nano-ESI-MS^E^) approach was used for qualitative proteomic analysis. A total of 100 and 121 proteins were identified from the midexponential and early stationary growth phases, respectively, of the *L. lactis* strain M4. During the exponential phase, the most important reaction was the generation of sufficient energy, whereas, in the early stationary phase, the metabolic energy pathways decreased and the biosynthesis of proteins became more important. Thus, the metabolism of the cells shifted from energy production in the exponential phase to the synthesis of macromolecules in the stationary phase. The resultant proteomes are essential in providing an improved view of the cellular machinery of *L. lactis* during the transition of growth phases and hence provide insight into various biotechnological applications.

## 1. Introduction

Lactic acid bacteria (LAB) are a heterogeneous group of Gram-positive bacteria that convert carbohydrates into lactic acid [1]. LAB are facultative anaerobic, nonspore forming rod- or coccus-shaped bacteria [2]. In nature, LAB are indigenous to food-related habitats such as plants and milk and occupy a niche on the mucosal surfaces of animals [3].* Lactococcus lactis *is the most studied species among LAB and has become the model bacterium for most LAB research in biotechnology. The two subspecies of* L. lactis *were initially designated as* Streptococcus lactis* and* Streptococcus cremoris *but were later reclassified as* L. lactis *ssp.* lactis* and* L. lactis* spp.* cremoris* [4].* L. lactis* has been exploited for a vast variety of biotechnological applications during the past few decades especially in the production of cheese. Its homofermentative nature and long history of safe usage in food preparation combined with its generally regarded as safe (GRAS) status have made* L. lactis* an economically important microorganism in the dairy food industry. In addition, a large amount of research on the metabolic engineering of* L. lactis* has been performed for the production of compounds such as diacetyl, alanine, and exopolysaccharides [5].* L. lactis* has also emerged as an efficient cell factory for the production of food ingredients [6], nutraceuticals [7], heterologous proteins [8, 9], and vaccine delivery platforms [10–12].

The genomes of several* L. lactis *strains have been completely sequenced, such as IL1403 [1], KF147 [13, 14], and CV56 [15] from* L. lactis *subsp.* lactis*, as well as strains such as MG1363 [16, 17] and SK11 [3] from* L. lactis* subsp.* Cremoris,* and the recently sequenced* L. lactis* IO-1 [18]. The availability of the complete genome sequence of multiple* L. lactis* strains enables the powerful application of proteomics to investigate the global cellular protein expression profiles of different* L. lactis* strains. Proteomics can be defined as the analysis of the whole-protein complement of the genome that is expressed by a cell or any biological sample at a given time under specific conditions [19]. Proteomics refers to large-scale protein studies with a particular emphasis on protein expression, structure, and function [20]. Although genomics and proteomics are complementary techniques, proteomics has the advantage of directly accessing biological processes at the protein level. Proteins are responsible for the structure, energy production, communication, movement, and division of all cells; thus, gaining a comprehensive understanding of proteins using systems biology is of utmost importance [21].

During the past decade, a number of proteomics studies on* L. lactis *have been performed. Initially, proteomics analyses were performed to set up reference maps [22, 23] and to better understand the cellular pathways of* L. lactis* related to important physiological processes and technological properties at the protein level [2]. Later, additional proteomics research on* L. lactis* focused on comparative expression studies [24–33] and stress-response studies [34–38] due to* L. lactis* economic importance. Novel applications for LAB as living vehicles for the targeting of antigens or therapeutics to the digestive mucosa are being developed and proteomics methods are being used to investigate and identify new markers of* L. lactis* adaptation to the mouse digestive tract [39, 40]. To date, only one proteomics study of* L. lactis* on differences between multiple strains [2] and posttranslational modifications [41] has been reported.

Conventional two-dimensional polyacrylamide gel electrophoresis (2-DE) coupled with mass spectrometry is commonly used for proteomics analysis. However, with the advancement of technology, high throughput instruments such as liquid chromatography tandem mass spectrometry (LC-MS/MS) have been invented to facilitate the research and development of protein studies. This gel-free, label-free, highly sensitive, and specific LC-MS/MS approach enables the effective separation of complex protein mixtures by eluting peptides and their corresponding fragment ions.

In the present work, the target microorganism was the Malaysian isolated* L. lactis *M4 dairy strain obtained from the milk of locally bred cows. The* L. lactis *strain M4 was identified as* L. lactis *spp.* lactis* based on the molecular analysis of the16S rRNA gene. It was chosen as our target microorganism because it has been proven to be a potential host for the expression of heterologous proteins [42]. The proteomes of the* L. lactis* M4 dairy strain at mid-exponential and early stationary growth phases were determined and the physiological changes between the growth phases were elucidated using highly sensitive and specific ultra-performance liquid chromatography nanoflow electrospray ionization tandem mass spectrometry (UPLC-nanoESI-MS/MS). The resultant proteomes are essential in providing an improved dynamic and global view of the* L. lactis* cellular machinery during growth phase transition. These proteomes may also have predictive value for the modeling of biological processes. They could also be utilized as a gold standard and make it possible to understand the differences in the protein expression patterns of local* L. lactis *strains between the growth phases, which in turn could provide insight into various applications such as the identification of target proteins that respond to alterations in diet or treatments, the development of effective cell factories for biorefinery through metabolic engineering, and the development of lactococcal expression systems in the future.

## 2. Materials and Methods

### 2.1. Bacterial Strains and Growth Conditions

The* Lactococcus lactis* M4 dairy strain (Malaysian isolated strain obtained from the milk of locally bred cows) was obtained courtesy of the Universiti Putra Malaysia (Serdang, Selangor, Malaysia). Frozen cell stocks of* L. lactis* M4 were streaked onto M17 plate supplemented with 0.5% glucose and grown at 30°C for overnight. Seed cultures were generated by transferring a single colony to a flask with M17 broth supplemented with 0.5% glucose. Seed cultures were incubated at 30°C without agitation for 16 h. The seed cultures were then diluted (2 mL into 200 mL) in M17 broth supplemented with 0.5% glucose. The cultures were incubated at 30°C without agitation until mid-exponential growth phase (OD_600_ ~ 0.8) or early stationary growth phase (OD_600_ ~ 1.8) and then harvested by centrifugation (7000 ×g for 15 minutes) using an Eppendorf 5810R centrifuge (Hamburg, Germany). The culture media were discarded, and the cells were frozen at −20°C until needed for protein extract preparation.

### 2.2. Protein Extraction and Trypsin Digestion

Protein extract was prepared using previously reported protocols [43, 44]. The cells were collected by centrifugation at 7000 ×g for 15 min, and the pellet was washed in phosphate buffer (1.24 g/L K_2_HPO_4_; 0.39 g/L KH_2_PO_4_; 8.8 g/L NaCl, pH 7.2). The pellet was then suspended in 1 mL of extraction buffer (0.7 M sucrose; 0.5 M Tris-HCl, pH7; 30 mM HCl; 50 mM EDTA; 0.1 M KCl; 40 mM DTT). The cell suspension was incubated for 15 min at room temperature and sonicated on ice (10 times for 10 s at 30 s intervals). The cell debris was removed by centrifugation at 10000 ×g for 30 min at 4°C. Protein concentration was estimated using the Quick Start Bradford Protein Assay (Bio-Rad, Hercules, CA, USA). The resulting protein extract was dispensed and kept at −20°C for subsequent analysis. To prepare the protein digest, approximately 250 *μ*g of total protein was denatured using 12.5 *μ*L of denaturing buffer (8 M urea and 25 mM NH_4_HCO_3_, pH 8.0). The mixture was then reduced with 10 mM DTT at 37°C for 1 h and alkylated with 50 mM iodoacetamide in the dark for 30 min. The urea concentration was reduced to 1 M by dilution with 50 mM NH_4_HCO_3_, pH 7.8. Proteolytic digestion was initiated by adding mass spectrometry grade Trypsin Gold (Promega, Madison, WI, USA) at a ratio of 1 : 50 (trypsin : protein) followed by incubation at 37°C overnight. Tryptic digestion was terminated by acidification with 0.1% trifluoroacetic acid. The tryptic peptide solution was kept at −80°C until further analysis.

### 2.3. Ultra-Performance Liquid Chromatography Tandem Mass Spectrometry (UPLC-MS/MS)

Tryptic digests of total protein obtained during both growth phases (mid-exponential and early stationary) were generated, separated, and analyzed using LC separation and MS analysis. Injections of 500 ng of total peptide were used in triplicate analyses (each biological replicate was subjected to two technical runs).

LC-nanoESI-MS^E^ (alternating low-energy MS and elevated energy MS^E^) analysis was performed using a SYNAPT G2 HDMS mass spectrometer equipped with a nanoACQUITY UPLC separation system. The aqueous mobile phase (mobile phase A) was water with 0.1% formic acid and the organic mobile phase (mobile phase B) was acetonitrile with 0.1% formic acid. Peptide separations were performed with an acetonitrile gradient in 0.1% formic acid (1–50% in 120 min). A total of 500 ng of peptides (partial loop, 1 *μ*L injection) was loaded onto a nanoACQUITY UPLC Symmetry C_18_, 180 × 20 mm trap column at 15 *μ*L/min for subsequent separation by LC-nanoESI using a nanoACQUITY UPLC BEH 130 C_18_, 75 *μ*m × 200 mm reverse phase column (Waters Corporation, Milford, MA, USA) with 1% mobile phase B at 0.25 *μ*L/min. Peptides were eluted from the column with a gradient of 1–50% mobile phase B over 90 min at 0.25 *μ*L/min followed by a 10 min rinse of 85% mobile phase B. The column was immediately reequilibrated under the initial conditions (1% mobile phase B) for 20 min. The lock mass, [Glu^1^]-fibrinopeptide at 100 fmol/*μ*L, was delivered from the fluidic system at 5 *μ*L/min to the reference sprayer of the NanoLockSpray source.

Mass spectrometry analysis of the separated tryptic digested peptides was performed using a SYNAPT G2 HDMS mass spectrometer. The instrument control and data acquisition were conducted using the MassLynx data system, version 4.1 (Waters Corporation, Milford, MA, USA). The electrospray voltage was set at 3 kV, the source temperature was 80°C, and the cone voltage was 40 V. For all experiments, the mass spectrometer was operated in resolution mode with a typical resolving power of at least 20000, and all analyses were performed using positive mode ESI using a NanoLockSpray source. The lock mass channel was sampled every 30 s. The spectral acquisition time in each mode was 1.0 s, and the mass spectra were acquired from* m*/*z* 50 to 2000. In low energy MS mode, data were collected at constant collision energy of 15 eV. In MS^E^ mode, the collision energy was ramped from 15 to 40 eV during each 1.0 s data collection cycle. One cycle of MS and MS^E^ data was acquired every 10.0 s.

### 2.4. Data Analysis

The continuum LCMS^E^ data were processed and searched using ProteinLynx Global Server (PLGS) version 2.4. Protein identifications were assigned by searching against the* Lactococcus* protein database available at UniProt. The search parameter values for each precursor and associated fragment ion were set by the software using the measured mass error and intensity error obtained from the processing of the raw continuum data. The mass error tolerance values were typically less than 5 ppm. Peptide identifications were restricted to tryptic peptides with no more than one missed cleavage. Cysteine carbamidomethylation was considered as a fixed peptide modification, whereas methionine oxidation, asparagines deamination, and glutamine deamination were considered as variable peptide modifications. Peptides with PLGS scores greater than 200 were considered for charting the proteome of the strain M4 at both growth phases (late-exponential and early stationary). PLGS score is calculated by the ProteinLynx Global Server (PLGS 2.4) software using a Monte Carlo algorithm to analyze all available mass spectrometry data and is a statistical measure of accuracy of identification. A high score implies greater confidence of protein identity [45]. Protein identification was manually validated for proteins with PLGS scores less than 200.

The identified proteins obtained from both growth phases (late-exponential and early stationary) were categorized based on their molecular functions. The functional classification of proteins was performed according to a previously published list of categories [1, 46]. The consensus lists of proteins were then analyzed to determine their cellular localization based on gene ontology using the STRAP software [47]. The proteomes were then compared and further analyzed for their association with different processes, networks, and pathway maps.

## 3. Results and Discussion

### 3.1. Identification of the Growth Phase-Dependent Proteomes

Our study presents the first comparative investigation of the Malaysian isolated* L. lactis *strain M4 proteome at two time points, the mid-exponential and early stationary growth phases, using a label-free shotgun proteomics approach.* L. lactis *M4 was grown at 30°C in M17 media. The optical density of cells at 600 nm was recorded hourly to construct the growth curve of* L. lactis* M4 and to identify the time points for the mid-exponential growth phase (OD_600_ ~ 0.8) and early stationary growth phase (OD_600_ ~ 1.8). To assure the comparable numbers of living cells for the subsequent extraction steps and comparative proteomics analysis, the OD_600 _reading was verified before the cells were harvested. These two growth phases were chosen because the production of organic acids (mainly lactic acid), ethanol, aroma compounds, bacteriocins, exopolysaccharides, and several enzymes is of importance to dairy and fermented food industry, and they are reported to be produced during the transition of these phases [48]. M17 medium was chosen as it is the commonly used growth medium for* L. lactis*. The protein extracts were proteolyzed and the tryptic digests were separated by ultra-performance liquid chromatography nanoflow electrospray ionization tandem mass spectrometry (UPLC-nanoESI-MS/MS).

**Figure 1:**
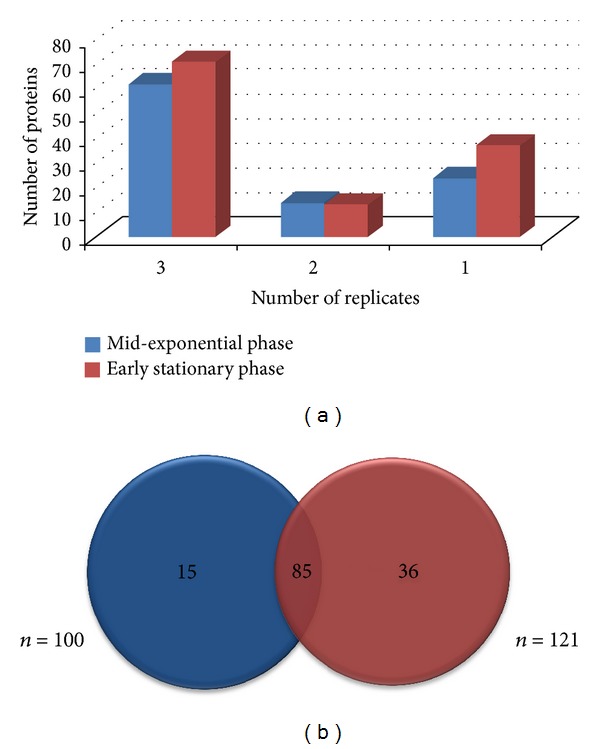
Comparison of the growth phase-dependent proteomes of* L. lactis *strain M4. Panel (a), the bar charts show the comparison of the number of proteins found in the biological replicates. Panel (b), the Venn diagrams show the comparison of the proteomes during the mid-exponential and early stationary growth phases.

Approximately 73158 and 75494 MS/MS peptide spectra were collected and analyzed for the mid-exponential and early stationary growth phases, respectively. The UPLC-nanoESI-MS/MS analysis yielded a total of 100 and 121 proteins from the mid-exponential and early stationary growth phases of* L. lactis* M4, respectively. The proteome dataset identified in the present study was small, corresponding to only 4.3–5.3% of all proteins predicted from the genome of* L. lactis* spp.* lactis* IL1403. However, this level of coverage is comparable to the proteome dataset of* Bifidobacterium longum* strain NCC2705, which was reported to correspond to 7.57% of the probable coding regions of* B. longum* NCC2705 [49, 50]. The search parameter values fixed in PLGS were stringent to increase the confidence level in protein identification in our qualitative proteomics study. This could be the main reason that low protein coverage was identified in our study. The low protein coverage may also be due to the lack of a published genome sequence specific to* L. lactis *strain M4. Protein coverage could be greatly improved if the genome of the Malaysian isolated* L. lactis* strain was completely sequenced and a multidimensional liquid chromatography tandem mass spectrometry approach was used. From the 100 and 121 proteins identified for the mid-exponential and early stationary growth phases, >50% of proteins were detected in three biological replicates(Figure 1(a)). When the proteomes from both growth phases were compared, 85 proteins were found to be present in both datasets (Figure 1(b)).

### 3.2. Functional Categorization of the Identified* L. lactis* Proteins

**Figure 2:**
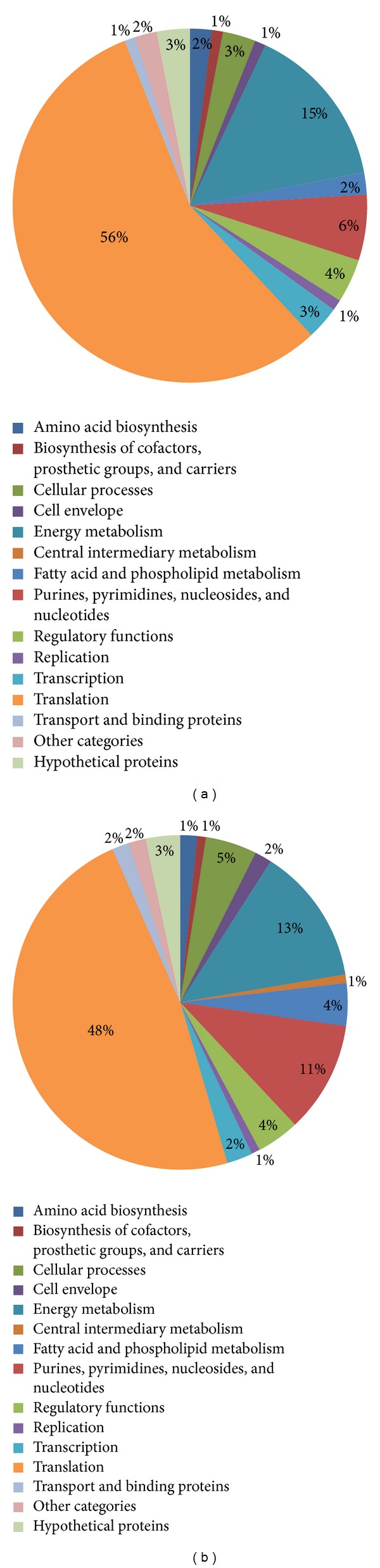
Functional attributions of the growth phase-dependent proteomes of* L. lactis* strain M4. Panel (a), the pie chart shows the functional attributions of* L. lactis* M4 proteome during the mid-exponential growth phase (OD_600_ ~ 0.8); Panel (b), the early stationary growth phase (OD_600_ ~ 1.8).

**Figure 3:**
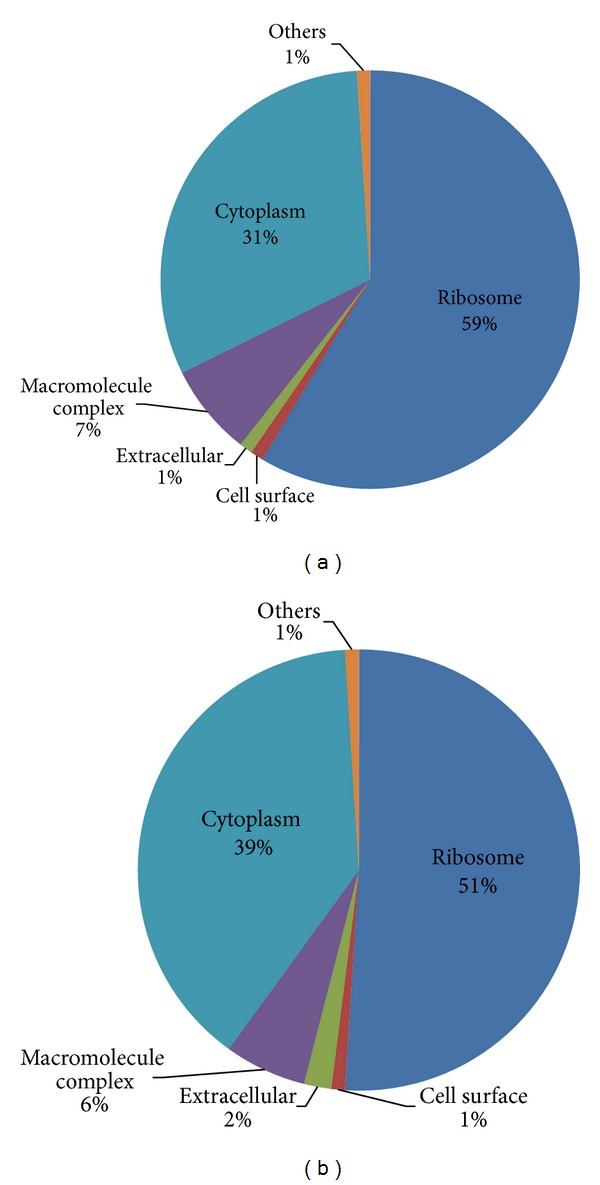
Cellular localization of the growth phase-dependent proteomes of* L. lactis *strain M4 based on gene ontology using STRAP software. Panel (a), the pie chart shows the cellular localization of* L. lactis *M4 proteome during the mid-exponential growth phase (OD_600_ ~ 0.8); Panel (b), the early stationary growth phase (OD_600_ ~ 1.8) based on gene ontology analysis.

The identified proteins from both growth phases were categorized according to their annotated functions (Figure 2, Table 1) and cellular localization based on gene ontology (Figure 3). The identified proteome dataset of* L. lactis* strain M4 was mainly associated with translation and energy metabolism, which comprised 56% and 15% of the identified proteins, respectively (Figure 2(a)), for the mid-exponential growth phase and 48% and 13% of the identified proteins, respectively, (Figure 2(b)) for the early stationary growth phase. The remaining identified proteins were categorized as purines, pyrimidines, nucleosides, and nucleotides or as being associated with regulatory functions, cellular processes, replication, transcription, amino acid biosynthesis, the biosynthesis of cofactors, prosthetic groups and carriers, cell envelope, fatty acid and phospholipid metabolism, transport and binding, hypothetical proteins, or as others (Figure 2).

The majority of the* L. lactis* strain M4 proteome for the mid-exponential growth phase was localized in the ribosome (59%), cytoplasm (31%), macromolecule complex (7%), cell surface (7%), extracellular space (7%), and other areas (7%) (Figure 3(a)). Similarly, the majority of the identified* L. lactis* strain M4 proteins from the early stationary growth phase were found to be localized in the ribosome (51%) followed by the cytoplasm (39%), macromolecule complex (6%), extracellular space (2%), cell surface (1%), and other areas (1%) (Figure 3(b)). Low percentage of extracellular proteins and cell surface proteins were identified in our study because the sample preparation was designed for the extraction of cytoplasmic proteins.

#### 3.2.1. Translation

Both the mid-exponential and early stationary growth phases* L. lactis* M4 proteome datasets were dominated by translation machinery proteins. A transcriptomic study of* L. lactis *MG1363 reported recently had also shown that genes involved in “translation, ribosomal structure, and biogenesis” were upregulated at the transition point from exponential to stationary growth [51]. Ribosomal proteins, which are some of the most highly expressed proteins in the cell, were identified in large numbers in the* L. lactis* M4 proteome for both growth phases. Based on the genome sequence of* L. lactis* subsp.* lactis* IL1403, 58 proteins were predicted to be ribosomal proteins [1]. In this study, there were 44 and 41 ribosomal proteins identified from the mid-exponential and early stationary growth phases, respectively.

In addition to ribosomal proteins, other proteins involved in translation, including peptidases (aminopeptidase C, aminopeptidase N, dipeptidase, peptidase T, proline dipeptidase, prolidase, and Xaa-Pro aminopeptidase), aminoacyl tRNA synthetases (arginyl-tRNA synthetase, isoleucyl-tRNA synthetase, phenylalanyl-tRNA synthetase beta chain, seryl-tRNA synthetase, threonyl-tRNA synthetase, and tyrosyl-tRNA synthetase), translation factors (elongation factors G, P, Ts, and Tu, translation initiation factor IF-1, and ribosome recycling factor), and proteins involved in modification (peptidyl-prolyl* cis-trans* isomerase), were identified in the proteomes from both growth phases. Previous studies have shown that a high expression of aminoacyl tRNA synthetases and ribosomal proteins is one of the characteristics of the early-exponential growth phase [52]; however, the expression level of these proteins could not be determined through our qualitative proteomics analysis. Nevertheless, it is important to note that a greater number of peptidases (aminopeptidase C, dipeptidase, peptidase T, prolidase, and Xaa-Pro aminopeptidase) were identified during the early stationary phase than during the mid-exponential phase. The expression of these peptidases only in the early stationary phase suggested that the selective degradation of proteins, peptides, or glycopeptides at this time is likely related to the cessation of growth.

#### 3.2.2. Energy Metabolism

Overall, 15% and 13% of the proteins from the mid-exponential and early stationary growth phases of the* L. lactis* M4 proteome datasets, respectively, were involved in energy metabolism. The majority of these expressed proteins were involved in glycolysis (enolase, fructose-bisphosphate aldolase, glyceraldehyde-3-phosphate dehydrogenase, L-lactate dehydrogenase, 6-phosphofructokinase, glucose-6-phosphate isomerase, phosphoglycerate kinase, phosphopyruvate hydratase, pyruvate kinase, triosephosphate isomerase, and 2,3-bisphosphoglycerate-dependent phosphoglycerate mutase) and the remaining proteins were involved in fermentation (alcohol-acetaldehyde dehydrogenase, formate acetyltransferase, and pyruvate-formate lyase), the pentose phosphate pathway (6-phosphogluconate dehydrogenase [decarboxylating]), pyruvate decarboxylation (pyruvate dehydrogenase complex E2 component), and the urea cycle (ornithine carbamoyltransferase). A greater number of proteins were involved in energy metabolism during mid-exponential growth than early stationary growth, which provides sufficient energy for growth [53]. Previous transcriptomic study also showed that genes involved in “energy production and conversion” respond during the exponential growth phase, but not so in the stationary phase [51]. The presence of L-lactate dehydrogenase indicates that lactic acid fermentation occurred in* L. lactis* M4 during both of the growth phases, and the presence of formate acetyltransferase indicates that mixed acid fermentation rather than homolactic fermentation occurred in* L. lactis *M4 during both of the growth phases [54].

#### 3.2.3. Purines, Pyrimidines, Nucleosides, and Nucleotides

In this class, 6 and 13 proteins from the mid-exponential and early stationary growth phases, respectively, were found to be involved inpurine ribonucleotide* de novo *biosynthesis (GMP synthase, IMP dehydrogenase, and adenylosuccinate synthase), pyrimidine ribonucleotide* de novo *biosynthesis (carbamoyl phosphate synthase, orotate phosphoribosyltransferase, and uridylate kinase), the salvage of nucleosides and nucleotides (adenylate kinase, phosphopentomutase, ribose-phosphate pyrophosphokinases, and uracil phosphoribosyltransferase), and sugar-nucleotide biosynthesis and interconversion (dTDP-glucose-4,6-dehydratase, glucose-1-phosphate thymidylyltransferase). A greater number of proteins involved in purines, pyrimidines, nucleosides, and nucleotides biosynthesis were expressed during the early stationary phase, indicating that the exhaustion of metabolites during this time necessitates the resynthesis of these proteins [52].

#### 3.2.4. Cellular Processes

In this study, 3% of proteins from the mid-exponential growth phase and 5% of proteins from the early stationary growth phase of the* L. lactis* M4 proteome were identified as chaperones or are involved in cellular processes such as cell division, detoxification, and protein and peptide secretion. Cell division protein GTPase FtsZ, chaperone protein DnaK, and trigger factor were detected for both growth phases. Previous studies have shown that FtsZ protein is more highly expressed during the beginning of the exponential phase for several bacteria species [55]. Additionally, DnaK protein has been reported to be expressed at different levels during different phases of growth which could reflect the adaptation of the cultures to changes in pH and the nutrient compositions [29]. Furthermore, trigger factor and DnaK protein cooperate in the folding of newly synthesized proteins [56]. Thus, our findings are relevant and well substantiated by these studies.

In addition to these findings, two proteins involved in detoxification, alkyl hydroperoxide reductase subunit C and superoxide dismutase [Mn], were identified only in the early stationary growth phase proteome for* L. lactis* M4. Superoxide dismutase [Mn] is only expressed under aerobic conditions [57] and is believed to be involved in the prevention of DNA damage [58]. Superoxide dismutase [Mn] converts oxygen radicals to hydrogen peroxide, which is important for the oxidative stress response. Studies have also shown that alkyl hydroperoxide reductase plays a pivotal role in oxidative stress resistance and acts on hydrogen peroxide [59, 60]. Reactive oxygen species (ROS) and reactive nitrogen intermediates (RNI) accumulate during the early stationary phase [61], and the expression of these detoxification proteins protects the cells from the oxidative stress caused by ROS and RNI.

#### 3.2.5. Regulatory Functions

Proteins involved in regulatory functions were identified during both growth phases of* L. lactis* M4 (4% of the proteome dataset for both phases). The function of transcriptional regulators such as transcriptional regulator CodY, transcriptional regulator CodZ, and bifunctional protein pyrR is to control the expression level of genes by modifying the rate of transcription during different growth phases. At different growth phases, two-component system regulators and transcriptional regulators are believed to act as a simple stimulus-response coupling mechanism to allow the cells to detect, act on, and adapt to various environmental changes, stresses, and growth conditions [62]. In addition, transcriptional regulator CodY was identified as the first global regulator with a low G+C content conserved in Gram-positive bacteria that regulates amino acid biosynthesis [63].

#### 3.2.6. Fatty Acid and Phospholipid Metabolism

A greater number of proteins involved in fatty acid and phospholipid metabolism were identified in the* L. lactis* M4 proteome during the early stationary growth phase (~4%) than during the mid-exponential growth phase (2%). These proteins include 3-oxoacyl-acyl carrier protein synthase 2,3-oxoacyl-acyl carrier protein reductase, the acetyl-CoA carboxylase biotin carboxylase subunit, the acyl carrier protein, and NADH-dependent enoyl-acyl carrier protein reductase. Enzymes involved in fatty acid and phospholipid metabolism are most likely expressed at a higher level during the early stationary growth phase due to the reinforcement of the cell membrane that occurs during the transition of growth phases where large numbers of phospholipids are needed [53].

#### 3.2.7. Replication and Transcription

A small number of proteins from the proteome datasets for both growth phases were involved in replication and transcription. Only one protein involved in the replication process, Hu-like DNA-binding protein, was identified in both growth phases. The transcriptional machinery proteins ATP-dependent RNA helicase, DNA-dependent-RNA polymerase alpha subunit, RNA polymerase beta subunit transcription antitermination protein, and transcription elongation factor GreA were identified in the proteome dataset from either the mid-exponential phase or early stationary growth phase.

#### 3.2.8. Amino Acid Biosynthesis

Two proteins involved in amino acid biosynthesis, glutamine synthetase and S-ribosylhomocysteine lyase, were identified in the* L. lactis* M4 proteome dataset for both the mid-exponential and early stationary growth phases. It was reported that glutamine synthetase (GS) coded by the* glnA* gene was highly expressed and increased in parallel to high consumption of glutamine. Glutamine is the most consumed amino acid in which it is used for synthesis of biomass proteins and it is the donor of amino groups in purine, pyrimidine, and amino sugar production pathways [28]. In addition, another study using 2-DE gel analysis reported an overexpression of GS when* L. lactis* NCDO763 was grown in milk medium. The authors suggested that this enzyme plays a vital role in the growth of* L. lactis* in milk medium and that glutamine production through GS activity is an important component of the metabolic adaptation of* L. lactis* tomilk medium. Overexpression was not observed in* L. lactis* NCDO763 grown in a rich medium such as M17 [27]. Nevertheless, from our proteome analysis, GM is believed to be synthesized by* L. lactis* M4 strain throughout its growth period as a part of its adaptation to varied culture conditions during different growth phases.

S-Ribosylhomocysteine lyase is encoded by* luxS* gene and is involved in the synthesis of autoinducer 2 (Al-2), which is secreted by bacteria during quorum sensing [64, 65]. Changes in the cell density during the transition of growth phases are likely to stimulate the expression of S-ribosylhomocysteine lyase and the subsequent synthesis of Al-2 in* L. lactis* M4.

#### 3.2.9. Biosynthesis of Cofactors, Prosthetic Groups, and Carriers

Only 1% of the proteome dataset of* L. lactis* M4 at both the mid-exponential growth phase and early stationary growth phase was associated with the biosynthesis of cofactors, prosthetic groups, and carriers. Enzyme 2-dehydropantoate-2-reductase was identified only during the mid-exponential growth phase. 2-Dehydropantoate-2-reductase is the second enzyme in the pantothenate biosynthesis pathway (vitamin B_5_) [66] and catalyzes the reduction of NADPH-dependent ketopantoate to pentanoate [67]. Cell division rates are increased when the bacterial growth enters the mid-exponential phase, and, thus, bacterial cells are required to synthesize large quantities of metabolites such as vitamins to support growth.

The thioredoxin system plays a crucial role in maintaining the redox conditions of the cells and protecting them against oxidative stress by reducing reactive oxygen species (ROS) and reactive nitrogen intermediates (RNI) through various mechanisms. ROS and RNI accumulate at the final stage of growth [61] and, therefore, thioredoxin was expressed by* L. lactis* M4 strain during the early stationary growth phase to maintain the homeostasis of the cell cytoplasm.

#### 3.2.10. Cell Envelope

Only 1% and 2% of the mid-exponential and early stationary growth phases of the* L. lactis* M4 proteome datasets, respectively, were categorized as being involved in cell envelope biosynthesis. UDP-N-acetylmuramoylalanine-D-glutamate ligase was found only in the early stationary growth phase proteome dataset; this protein belongs to the murCDEF family and is involved in peptidoglycan synthesis. The induction of enzymes involved in the synthesis of cell wall structures during the stationary phase is thought to strengthen the cell wall and maintain the bacterial morphology [53]. UDP-glucose-1-phosphate uridylyltransferase (GalU) was identified in the proteome datasets for both phases. GalU is needed for capsular polysaccharide biosynthesis [68], in which it catalyzes the reversible formation of uridine-diphosphate glucose (UDP-Glc) and inorganic pyrophosphate (PPi) from uridine-3-phosphate (UTP) and glucose-1-phosphate (Glc-1P). UDP-Glc is a substrate for the synthesis of UDP-glucuronic acid and it is also important for the interconversion of galactose and glucose via the Leloir pathway.

#### 3.2.11. Transport and Binding Proteins

Only 1% and 2% of the mid-exponential and early stationary growth phases proteome datasets of* L. lactis* M4, respectively, were found to be involved in the transport and binding of proteins. Phosphoenolpyruvate protein phosphotransferase PtsI was identified in the proteome datasets for both growth phases. PTS systems contain phase-dependent proteins with the highest expression occurring during the exponential growth phase and decreased expression occurring progressively thereafter. However, continuous PtsI expression has been shown to occur throughout both growth phases [52], which explains the detection of Ptsl in the* L. lactis* M4 proteome dataset for both growth phases. Maltose ABC transporter substrate-binding protein, on the other hand, was found only in the early stationary growth phase proteome dataset. The appearance of maltose ABC transporter substrate-binding protein during the early stationary phase suggests that* L. lactis* shifted from utilizing of glucose as a carbon source to alternative carbon sources such as maltose due to the decreasing glucose content in the medium during the stationary growth phase.

#### 3.2.12. Central Intermediary Metabolism

S-Adenosylmethionine synthase was the only protein identified in the* L. lactis* M4 proteome during the early stationary growth phase that was classified under the category of central intermediary metabolism. S-Adenosylmethionine synthase is involved in the biosynthesis of S-adenosyl-L-methionine and converts L-methionine and ATP to S-adenosyl-L-methionine which is required for methyltransferase reactions in the cell and for polyamine biosynthesis [69].

#### 3.2.13. Other Categories

A small number of proteins implicated in the stress response were identified in the* L. lactis* M4 proteome for both growth phases (~2%), including ClpB protein, cold shock protein E, and stress response protein E. Usually, stress proteins are induced and expressed more highly during stationary phase due to unfavorable conditions such as diminishing nutrient levels and high concentrations of lactic acid in the medium [53]. However, the expression pattern could not be observed in our qualitative proteomics study.

#### 3.2.14. Hypothetical Proteins

Approximately 3% of the* L. lactis* M4 proteome datasets for the mid-exponential and early stationary growth phases were identified as hypothetical proteins. These are putative proteins that have not been purified and experimentally identified.

## 4. Conclusions

The present highly sensitive label-free qualitative shotgun comparative proteome analysis is the first study of the phase-dependent proteomes of* L. lactis *strain M4. A qualitative difference between the phase-dependent proteomes of* L. lactis* M4 in terms of the absolute numbers of proteins identified was studied, the identified proteins were categorized according to annotated functions, and the proteomes were compared and analyzed. In summary, the most important reaction occurring during the exponential phase was the generation of sufficient energy, whereasthe metabolic energy pathways decreased and the biosynthesis of proteins became more important during the early stationary phase. Thus, the metabolism of the cells shifted from energy production in the exponential phase to the synthesis of macromolecules in the stationary phase. Additionally, the accumulation of ROS and RNI in the late stage of growth induced the expression of proteins that can protect the cells against oxidative stress. Many stress proteins were also strongly induced in the stationary phase due to unfavorable conditions such as high acid and diminished nutrient levels.

In the future, more advanced proteomics platforms, such as multidimensional liquid chromatography tandem mass spectrometry, can be used to increase the protein coverage and to reveal the quantitative differences between the* L. lactis* M4 proteome at different growth phases.

## Supplementary Material

Supplementary Table: PLGS score, number of peptides, protein coverage and number of replicates for the growth phase-dependent proteomes of *L. lactis* strain M4.

## Figures and Tables

**Table 1:** List of the proteins identified at the midexponential (ME) and early stationary (ES) growth phases of *L.  lactis *strain M4.

Gene name	Protein name	ME	ES
	*Amino acid biosynthesis *		
glnA	Glutamine synthetase	•	•
luxS	S-Ribosylhomocysteine lyase	•	•
	Biosynthesis of cofactors, prosthetic groups, and carriers		
panE	2-Dehydropantoate-2-reductase	•	
trxH	Thioredoxin H-type		•
	*Cellular processes *		
ahpC	Alkyl hydroperoxide reductase subunit C		•
ftsZ	Cell division protein GTPase FtsZ	•	•
divIVA	Cell division initiation protein DivIVA		•
dnaK	Chaperone protein DnaK	•	•
sodA	Superoxide dismutase [Mn]		•
tig	Trigger factor	•	•
	*Cell envelope *		
murD	UDP-N-acetylmuramoylalanine-D-glutamate ligase		•
hasC/galU	UDP-glucose-1-phosphate uridylyltransferase	•	•
	*Energy metabolism *		
pmg	2,3-Bisphosphoglycerate-dependent phosphoglycerate mutase	•	•
pfk	6-Phosphofructokinase	•	•
gnd	6-Phosphogluconate dehydrogenase decarboxylating	•	•
adhE	Alcohol-acetaldehyde dehydrogenase	•	•
enoA	Enolase	•	•
pfl	Formate acetyltransferase	•	•
fbaA	Fructose-bisphosphate aldolase	•	•
pgiA	Glucose-6-phosphate isomerase	•	•
gapB	Glyceraldehyde-3-phosphate dehydrogenase	•	•
ldhA	L-Lactate dehydrogenase 1	•	•
arcB	Ornithine carbamoyltransferase 2	•	
pgk	Phosphoglycerate kinase	•	•
enoB	Phosphopyruvate hydratase	•	•
pdhC	Pyruvate dehydrogenase complex E2 component		•
LACR_0691	Pyruvate-formate lyase		•
pyk	Pyruvate kinase	•	•
tpiA	Triosephosphate isomerase	•	•
	*Central intermediary metabolism *		
metK	S-Adenosylmethionine synthase		•
	*Fatty acid and phospholipid metabolism *		
fabF	3-Oxoacyl-acyl carrier protein synthase 2	•	•
fabG1	3-Oxoacyl-acyl carrier protein reductase		•
accB	Acetyl-CoA carboxylase biotin carboxylase subunit		•
acpA	Acyl carrier protein	•	•
fabI	NADH-dependent enoyl-acyl carrier protein reductase		•
	*Purines, pyrimidines, nucleosides, and nucleotides *		
adk	Adenylate kinase	•	•
purA	Adenylosuccinate synthase		•
carB	Carbamoyl phosphate synthase large chain		•
rmlB	dTDP-glucose-4,6-dehydratase		•
rmlA	Glucose-1-phosphate thymidylyltransferase		•
guaA	GMP synthase glutamine hydrolyzing	•	•
guaB	Inosine-5-monophosphate dehydrogenase	•	•
pyrE	Orotate phosphoribosyltransferase	•	•
deoB	Phosphopentomutase	•	•
prsA	Ribose-phosphate pyrophosphokinase		•
prsB	Ribose-phosphate pyrophosphokinase		•
upp	Uracil phosphoribosyltransferase	•	•
pyrH	Uridylate kinase		•
	*Regulatory functions *		
pyrR	Bifunctional protein pyrR	•	•
typA	GTP-binding protein TypA/BipA	•	•
codY	Transcriptional regulator	•	•
codZ	Transcriptional regulator		•
llrC	Two-component system regulator	•	•
	*Replication *		
hslA	Hu-like DNA-binding protein	•	•
	*Transcription *		
rheA	ATP-dependent RNA helicase	•	
rpoA	DNA-dependent RNA polymerase alpha subunit	•	•
rpoB	DNA-dependent RNA polymerase beta subunit		•
nusG	Transcription antitermination protein	•	
greA	Transcription elongation factor GreA		•
	*Translation *		
rpsA	30S ribosomal protein S1	•	•
rpsB	30S ribosomal protein S2	•	•
rpsC	30S ribosomal protein S3	•	•
rpsD	30S ribosomal protein S4	•	•
rpsE	30S ribosomal protein S5	•	•
rpsF	30S ribosomal protein S6	•	•
rpsG	30S ribosomal protein S7	•	•
rpsH	30S ribosomal protein S8	•	•
rpsI	30S ribosomal protein S9	•	•
rpsJ	30S ribosomal protein S10	•	•
rpsL	30S ribosomal protein S12	•	•
rpsM	30S ribosomal protein S13	•	•
rpsN	30S ribosomal protein S14	•	•
rpsP	30S ribosomal protein S16	•	•
rpsR	30S ribosomal protein S18	•	•
rpsS	30S ribosomal protein S19	•	
rpsT	30S ribosomal protein S20	•	•
rpsU	30S ribosomal protein S21	•	
rplA	50S ribosomal protein L1	•	•
rplB	50S ribosomal protein L2	•	•
rplC	50S ribosomal protein L3	•	•
rplD	50S ribosomal protein L4	•	•
rplE	50S ribosomal protein L5	•	•
rplF	50S ribosomal protein L6	•	•
rplL	50S ribosomal protein L7/L12	•	•
rplJ	50S ribosomal protein L10	•	•
rplK	50S ribosomal protein L11	•	•
rplM	50S ribosomal protein L13	•	•
rplO	50S ribosomal protein L15	•	•
rplP	50S ribosomal protein L16	•	•
rplQ	50S ribosomal protein L17	•	•
rplR	50S ribosomal protein L18	•	•
rplS	50S ribosomal protein L19	•	•
rplT	50S ribosomal protein L20		•
rplU	50S ribosomal protein L21	•	•
rplV	50S ribosomal protein L22	•	•
rplW	50S ribosomal protein L23	•	•
rpmA	50S ribosomal protein L27	•	
rpmB	50S ribosomal protein L28	•	•
rpmC	50S ribosomal protein L29	•	•
rpmD	50S ribosomal protein L30	•	•
rpmE2	50S ribosomal protein L31 type B	•	•
rpmF	50S ribosomal protein L32	•	•
rpmG1	50S ribosomal protein L33 1	•	
rpmJ	50S ribosomal protein L36	•	•
pepC	Aminopeptidase C		•
pepN	Aminopeptidase N	•	•
argS	Arginyl-tRNA synthetase	•	•
pepV	Dipeptidase		•
fusA	Elongation factor G	•	•
efp	Elongation factor P		•
tsf	Elongation factor Ts	•	•
tuf	Elongation factor Tu	•	•
ileS	Isoleucyl-tRNA synthetase	•	
pepT	Peptidase T		•
ppiB	Peptidyl-prolyl *cis-trans* isomerase	•	•
pheT	Phenylalanyl-tRNA synthetase beta chain		•
pepQ	Proline dipeptidase	•	•
pepO	Prolidase		•
frr	Ribosome recycling factor	•	•
serS	Seryl-tRNA synthetase	•	•
thrS	Threonyl-tRNA synthetase	•	
infA	Translation initiation factor IF-1		•
tyrS	Tyrosyl-tRNA synthetase	•	
LACR_1813	Xaa-Pro aminopeptidase		•
	*Transport and binding proteins *		
ptsl	Phosphoenolpyruvate protein phosphotransferase	•	•
malE	Maltose ABC transporter substrate binding protein		•
	*Other categories *		
clpB	ClpB protein		•
cspE	Cold shock protein E	•	•
grpE	Stress response protein E	•	
	*Hypothetical proteins *		
yhjA	General stress protein, CsbD superfamily		•
ytjD	Nitroreductase family protein		•
llmg_1773	Putative uncharacterized protein	•	•
llmg_2049	Putative uncharacterized protein	•	
SA8A11-2	SA8A11-2 protein		•
LACR_1462	UDP-glucose pyrophosphorylase	•	

## References

[1] Bolotin A, Wincker P, Mauger S (2001). The complete genome sequence of the lactic acid bacterium *Lactococcus lactis* ssp. *lactis* IL1403. *Genome Research*.

[2] Guillot A, Gitton C, Anglade P, Mistou M-Y (2003). Proteomic analysis of *Lactococcus lactis*, a lactic acid bacterium. *Proteomics*.

[3] Makarova K, Slesarev A, Wolf Y (2006). Comparative genomics of the lactic acid bacteria. *Proceedings of the National Academy of Sciences of the United States of America*.

[4] Schleifer KH, Kraus J, Dvorak C (1985). Transfer of *Streptococcus lactis* and related Streptococci to the genus *Lactococcus* gen. nov. *Systematic and Applied Microbiology*.

[5] Kleerebezemab M, Hols P, Hugenholtz J (2000). Lactic acid bacteria as a cell factory: rerouting of carbon metabolism in *Lactococcus lactis* by metabolic engineering. *Enzyme and Microbial Technology*.

[6] Jeroen H (2008). The lactic acid bacterium as a cell factory for food ingredient production. *International Dairy Journal*.

[7] Hugenholtz J, Sybesma W, Groot MN (2002). Metabolic engineering of lactic acid bacteria for the production of nutraceuticals. *Antonie van Leeuwenhoek*.

[8] le Loir Y, Azevedo V, Oliveira SC (2005). Protein secretion in *Lactococcus lactis*: an efficient way to increase the overall heterologous protein production. *Microbial Cell Factories*.

[9] Morello E, Bermúdez-Humarán LG, Llull D (2008). *Lactococcus lactis*, an efficient cell factory for recombinant protein production and secretion. *Journal of Molecular Microbiology and Biotechnology*.

[10] Bahey-el-Din M, Griffin BT, Gahan CGM (2008). Nisin inducible production of listeriolysin O in *Lactococcus lactis* NZ9000. *Microbial Cell Factories*.

[11] Raha AR, Varma NRS, Yusoff K, Ross E, Foo HL (2005). Cell surface display system for *Lactococcus lactis*: a novel development for oral vaccine. *Applied Microbiology and Biotechnology*.

[12] Steidler L (2001). *Lactococcus lactis*, a tool for the delivery of therapeutic proteins treatment of IBD. *TheScientificWorldJournal*.

[13] Siezen RJ, Bayjanov J, Renckens B (2010). Complete genome sequence of *Lactococcus lactis* subsp. *lactis* KF147, a plant-associated lactic acid bacterium. *Journal of Bacteriology*.

[14] Siezen RJ, Starrenburg MJC, Boekhorst J, Renckens B, Molenaar D, van Hylckama Vlieg JET (2008). Genome-scale genotype-phenotype matching of two *Lactococcus lactis* isolates from plants identifies mechanisms of adaptation to the plant niche. *Applied and Environmental Microbiology*.

[15] Gao Y, Lu Y, Teng K-L (2011). Complete genome sequence of *Lactococcus lactis* subsp. *lactis* CV56, a probiotic strain isolated from the vaginas of healthy women. *Journal of Bacteriology*.

[16] Wegmann U, O’Connell-Motherway M, Zomer A (2007). Complete genome sequence of the prototype lactic acid bacterium *Lactococcus lactis* subsp. *cremoris* MG1363. *Journal of Bacteriology*.

[17] Linares DM, Kok J, Poolman B (2010). Genome sequences of *Lactococcus lactis* MG1363 (revised) and NZ9000 and comparative physiological studies. *Journal of Bacteriology*.

[18] Kato H, Shiwa Y, Oshima K (2012). Complete genome sequence of *Lactococcus lactis* IO-1, a lactic acid bacterium that utilizes xylose and produces high levels of l-lactic acid. *Journal of Bacteriology*.

[19] Graham RLJ, Graham C, McMullan G (2007). Microbial proteomics: a mass spectrometry primer for biologists. *Microbial Cell Factories*.

[20] Zhang X, Fang A, Riley CP, Wang M, Regnier FE, Buck C (2010). Multi-dimensional liquid chromatography in proteomics—a review. *Analytica Chimica Acta*.

[21] Karpievitch YV, Polpitiya AD, Anderson GA, Smith RD, Dabney AR (2010). Liquid chromatography mass spectrometry-based proteomics: biological and technological aspects. *The Annals of Applied Statistics*.

[22] Anglade P, Demey E, Labas V, le Caer JP, Chich JF (2000). Towards a proteomic map of *Lactococcus lactis* NCDO 763. *Electrophoresis*.

[23] Drews O, Reil G, Parlar H, Görg A (2004). Setting up standards and a reference map for the alkaline proteome of the Gram-positive bacterium *Lactococcus lactis*. *Proteomics*.

[24] Mazzoli R, Pessione E, Dufour M (2010). Glutamate-induced metabolic changes in *Lactococcus lactis* NCDO 2118 during GABA production: combined transcriptomic and proteomic analysis. *Amino Acids*.

[25] Palmfeldt J, Levander F, Hahn-Hägerdal B, James P (2004). Acidic proteome of growing and resting *Lactococcus lactis* metabolizing maltose. *Proteomics*.

[26] Willemoës M, Kilstrup M, Roepstorff P, Hammer K (2002). Proteome analysis of a *Lactococcus lactis* strain overexpressing gapA suggests that the gene product is an auxiliary glyceraldehyde 3-phosphate dehydrogenase. *Proteomics*.

[27] Gitton C, Meyrand M, Wang J (2005). Proteomic signature of *Lactococcus lactis* NCDO763 cultivated in milk. *Applied and Environmental Microbiology*.

[28] Lahtvee P-J, Adamberg K, Arike L, Nahku R, Aller K, Vilu R (2011). Multi-omics approach to study the growth efficiency and amino acid metabolism in *Lactococcus lactis* at various specific growth rates. *Microbial Cell Factories*.

[29] Larsen N, Boye M, Siegumfeldt H, Jakobsen M (2006). Differential expression of proteins and genes in the lag phase of *Lactococcus lactis* subsp. *lactis* grown in synthetic medium and reconstituted skim milk. *Applied and Environmental Microbiology*.

[30] Magnani D, Barré O, Gerber SD, Solioz M (2008). Characterization of the CopR regulon of *Lactococcus lactis* IL1403. *Journal of Bacteriology*.

[31] Marreddy RKR, Geertsma ER, Permentier HP, Pinto JPC, Kok J, Poolman B (2010). Amino acid accumulation limits the overexpression of proteins in *Lactococcus lactis*. *PLoS ONE*.

[32] Vido K, le Bars D, Mistou M-Y, Anglade P, Gruss A, Gaudu P (2004). Proteome analyses of heme-dependent respiration in *Lactococcus lactis*: involvement of the proteolytic system. *Journal of Bacteriology*.

[33] Beyer NH, Roepstorff P, Hammer K, Kilstrup M (2003). Proteome analysis of the purine stimulon from *Lactococcus lactis*. *Proteomics*.

[34] Budin-Verneuil A, Pichereau V, Auffray Y, Ehrlich D, Maguin E (2007). Proteome phenotyping of acid stress-resistant mutants of *Lactococcus lactis* MG1363. *Proteomics*.

[35] Budin-Verneuil A, Pichereau V, Auffray Y, Ehrlich DS, Maguin E (2005). Proteomic characterization of the acid tolerance response in *Lactococcus lactis* MG1363. *Proteomics*.

[36] Zhang Y, Zhang Y, Zhu Y, Mao S, Li Y (2010). Proteomic analyses to reveal the protective role of glutathione in resistance of *Lactococcus lactis* to osmotic stress. *Applied and Environmental Microbiology*.

[37] Cesselin B, Ali D, Gratadoux J-J (2009). Inactivation of the *Lactococcus lactis* high-affinity phosphate transporter confers oxygen and thiol resistance and alters metal homeostasis. *Microbiology*.

[38] García-Quintáns N, Repizo G, Martín M, Magni C, López P (2008). Activation of the diacetyl/acetoin pathway in *Lactococcus lactis* subsp. *lactis* bv. diacetylactis CRL264 by acidic growth. *Applied and Environmental Microbiology*.

[39] Beganović J, Guillot A, van de Guchte M (2010). Characterization of the insoluble proteome of *Lactococcus lactis* by SDS-PAGE LC-MS/MS leads to the identification of new markers of adaptation of the bacteria to the mouse digestive tract. *Journal of Proteome Research*.

[40] Roy K, Meyrand M, Corthier G, Monnet V, Mistou M-Y (2008). Proteomic investigation of the adaptation of *Lactococcus lactis* to the mouse digestive tract. *Proteomics*.

[41] Soufi B, Gnad F, Jensen PR (2008). The Ser/Thr/Tyr phosphoproteome of *Lactococcus lactis* IL1403 reveals multiply phosphorylated proteins. *Proteomics*.

[42] Noreen N, Hooi WY, Baradaran A (2011). *Lactococcus lactis* M4, a potential host for the expression of heterologous proteins. *Microbial Cell Factories*.

[43] Silva JC, Denny R, Dorschel C (2006). Simultaneous qualitative and quantitative analysis of the *Escherichia coli* proteome: a sweet tale. *Molecular & Cellular Proteomics*.

[44] Soares MR, Facincani AP, Ferreira RM (2010). Proteome of the phytopathogen *Xanthomonas citri* subsp. *citri*: a global expression profile. *Proteome Science*.

[45] Rosenegger D, Wright C, Lukowiak K (2010). A quantitative proteomic analysis of long-term memory. *Molecular Brain*.

[46] Bolotin A, Mauger S, Malarme K, Ehrlich SD, Sorokin A (1999). Low-redundancy sequencing of the entire *Lactococcus lactis* IL1403 genome. *Antonie van Leeuwenhoek*.

[47] Bhatia VN, Perlman DH, Costello CE, McComb ME (2009). Software tool for researching annotations of proteins: open-source protein annotation software with data visualization. *Analytical Chemistry*.

[48] de Vuyst L, Leroy F (2007). Bacteriocins from lactic acid bacteria: production, purification, and food applications. *Journal of Molecular Microbiology and Biotechnology*.

[49] Guillaume E, Berger B, Affolter M, Kussmann M (2009). Label-free quantitative proteomics of two *Bifidobacterium longum* strains. *Journal of Proteomics*.

[50] Schell MA, Karmirantzou M, Snel B (2002). The genome sequence of *Bifidobacterium longum* reflects its adaptation to the human gastrointestinal tract. *Proceedings of the National Academy of Sciences of the United States of America*.

[51] de Jong A, Hansen ME, Kuipers OP, Kilstrup M, Kok J (2013). The transcriptional and gene regulatory network of *Lactococcus lactis* MG1363 during growth in milk. *PLoS ONE*.

[52] Koistinen KM, Plumed-Ferrer C, Lehesranta SJ, Kärenlampi SO, von Wright A (2007). Comparison of growth-phase-dependent cytosolic proteomes of two *Lactobacillus plantarum* strains used in food and feed fermentations. *FEMS Microbiology Letters*.

[53] Cohen DPA, Renes J, Bouwman FG (2006). Proteomic analysis of log to stationary growth phase *Lactobacillus plantarum* cells and a 2-DE database. *Proteomics*.

[54] Garrigues C, Loubiere P, Lindley ND, Cocaign-Bousquet M (1997). Control of the shift from homolactic acid to mixed-acid fermentation in *Lactococcus lactis*: predominant role of the NADH/NAD+ ratio. *Journal of Bacteriology*.

[55] Weart RB, Levin PA (2003). Growth rate-dependent regulation of medial FtsZ ring formation. *Journal of Bacteriology*.

[56] Deuerling E, Schulze-Specking A, Tomoyasu T, Mogk A, Bukau B (1999). Trigger factor and DnaK cooperate in folding of newly synthesized proteins. *Nature*.

[57] Touati D (1988). Transcriptional and posttranscriptional regulation of manganese superoxide dismutase biosynthesis in *Escherichia coli*, studied with operon and protein fusions. *Journal of Bacteriology*.

[58] Hopkin KA, Papazian MA, Steinman HM (1992). Functional differences between manganese and iron superoxide dismutases in *Escherichia coli* K-12. *The Journal of Biological Chemistry*.

[59] Sanders JW, Leenhouts KJ, Haandrikman AJ, Venema G, Kok J (1995). Stress response in *Lactococcus lactis*: cloning, expression analysis, and mutation of the lactococcal superoxide dismutase gene. *Journal of Bacteriology*.

[60] Wasim M, Bible AN, Xie Z, Alexandre G (2009). Alkyl hydroperoxide reductase has a role in oxidative stress resistance and in modulating changes in cell-surface properties in *Azospirillum brasilense* Sp245. *Microbiology*.

[61] Soares NC, Cabral MP, Gayoso C (2010). Associating growth-phase-related changes in the proteome of *Acinetobacter baumannii* with increased resistance to oxidative stress. *Journal of Proteome Research*.

[62] Skerker JM, Prasol MS, Perchuk BS, Biondi EG, Laub MT (2005). Two-component signal transduction pathways regulating growth and cell cycle progression in a bacterium: a system-level analysis. *PLoS Biology*.

[63] Guédon E, Sperandio B, Pons N, Ehrlich SD, Renault P (2005). Overall control of nitrogen metabolism in *Lactococcus lactis* by CodY, and possible models for CodY regulation in Firmicutes. *Microbiology*.

[64] Cao J-G, Meighen EA (1989). Purification and structural identification of an autoinducer for the luminescence system of *Vibrio harveyi*. *The Journal of Biological Chemistry*.

[65] Taga ME, Semmelhack JL, Bassler BL (2001). The LuxS-dependent autoinducer Al-2 controls the expression of an ABC transporter that functions in Al-2 uptake in *Salmonella typhimurium*. *Molecular Microbiology*.

[66] Webb ME, Smith AG, Abell C (2004). Biosynthesis of pantothenate. *Natural Product Reports*.

[67] Zheng R, Blanchard JS (2000). Identification of active site residues in *E. coli* ketopantoate reductase by mutagenesis and chemical rescue. *Biochemistry*.

[68] Bonofiglio L, García E, Mollerach M (2005). Biochemical characterization of the pneumococcal glucose 1-phosphate uridylyltransferase (GalU) essential for capsule biosynthesis. *Current Microbiology*.

[69] Roje S (2006). S-Adenosyl-l-methionine: beyond the universal methyl group donor. *Phytochemistry*.

