# Supplementary material for: Growth Phase-Dependent Proteomes of the Malaysian Isolated Lactococcus lactis Dairy Strain M4 Using Label-Free Qualitative Shotgun Proteomics Analysis
Source: ScientificWorldJournal. 2014 Mar 25;2014:642891. doi: 10.1155/2014/642891 (PMC3984853; doi:10.1155/2014/642891)
Supplement: Supplementary file 1 — Supplementary Table: PLGS score, number of peptides, protein coverage and number of replicates for the growth phase-dependent proteomes of L. lactis strain M4. [file 642891.f1.docx]

| **TABLE S1** PLGS score, number of peptides, protein coverage and number of replicate for the growth-phase-dependent proteomes of *L. lactis* M4 strain  **Gene Name** | **Protein Name** | **PLGS**  **Score***  **(a, b)** | **Number**  **Of peptides***  **(a^1^, a^2^, b^1^, b^2^)** | **Protein**  **Coverage**  **(%) ***  **(a, b)** | **Number of**  **Replicate***  **(a, b)** |
| --- | --- | --- | --- | --- | --- |
|  | **Amino Acid Biosynthesis** |  |  |  |  |
| glnA | Glutamine synthetase | 1010.429,  1431.045 | 11, 50,  13, 76 | 33.86,  39.69 | 3, 3 |
| luxS | S-ribosylhomocysteine lyase | 619.3924,  398.2625 | 5, 14,  7, 22 | 29.75,  40.51 | 2, 3 |
|  | **Biosynthesis of Cofactors, Prosthetic Groups and Carriers** |  |  |  |  |
| panE | 2-dehydropantoate-2-reductase | 102.6433 | 5, 12 | 18.27 | 1 |
| trxH | Thioredoxin H-type | 296.7217 | 2, 9 | 26.67 | 1 |
|  | **Cellular Processes** |  |  |  |  |
| ahpC | Alkyl hydroperoxide reductase subunit C | 291.386 | 5, 19 | 39.57 | 1 |
| ftsZ | Cell division protein GTPase FtsZ | 538.4547,  615.2121 | 9, 32,  13, 43 | 24.46,  31.98 | 3, 3 |
| divIVA | Cell division initiation protein DivIVA | 250.145 | 6, 19 | 26.38 | 1 |
| dnaK | Chaperone protein DnaK | 1339.322,  683.4757 | 22, 96,  17, 74 | 38.06,  29.32 | 3, 3 |
| sodA | Superoxide dismutase [Mn] | 143.8565 | 5, 18 | 25.73 | 1 |
| tig | Trigger Factor | 3385.021,  2277.735 | 17, 112,  15,96 | 44.26,  40.05 | 3, 3 |
|  | **Cell Envelope** |  |  |  |  |
| murD | UDP-N-acetylmuramoylalanine-D-glutamate ligase | 308.5772 | 6, 14 | 6.77 | 1 |
| hasC/galU | UDP-glucose-1-phosphate uridylyltransferase | 406.3463,  546.9153 | 4, 18,  8, 32 | 24.28,  39.62 | 2, 3 |
|  | **Energy Metabolism** |  |  |  |  |
| pmg | 2,3-bisphosphoglycerate-dependent phosphoglycerate mutase | 2301.835, 2747.268 | 8, 33, 3,20 | 30.04, 18.03 | 3, 3 |
| pfk | 6-phosphofructokinase | 243.3102,  356.929 | 8, 34,  6, 25 | 15.29,  10 | 1, 2 |
| gnd | 6-phosphogluconate dehydrogenase decarboxylating | 897.8368,  514.4185 | 11, 40,  8, 28 | 17.8,  13.56 | 3, 3 |
| adhE | Alcohol-acetaldehyde dehydrogenase | 357.2367,  267.0663 | 15, 51,  24, 68 | 20.04,  24.47 | 2, 3 |
| enoA | Enolase | 32995.68,  27334.74 | 35, 231,  29, 190 | 54.73,  59.35 | 3, 3 |
| pfl | Formate acetyltransferase | 180.8088,  276.4884 | 12, 48,  15, 54 | 13.47,  20.58 | 2, 3 |
| fbaA | Fructose-bisphosphate aldolase | 1768.397,  1894.372 | 19, 92,  20, 112 | 55.37,  61.74 | 3, 3 |
| pgiA | Glucose-6-phosphate isomerase | 195.4196,  221.1219 | 6, 23,  10, 35 | 16.74,  27.9 | 1, 3 |
| gapB | Glyceraldehyde-3-phosphate dehydrogenase | 18567.38,  18526.32 | 38, 273,  38, 241 | 48.51,  51.49 | 3, 3 |
| ldhA | L-lactate dehydrogenase 1 | 161.0288,  1176.832 | 9, 34, 14, 60 | 34.46,  39.38 | 2, 2 |
| arcB | Ornithine carbamoyltransferase 2 | 285.6968 | 7, 22 | 22.6 | 1 |
| pgk | Phosphoglycerate kinase | 8305.882,  4379.465 | 25, 190,  20, 126 | 39.7,  41.96 | 3, 3 |
| enoB | Phosphopyruvate hydratase | 18555.92,  17601.36 | 8, 42,  7, 34 | 65.79,  63.16 | 3, 3 |
| pdhC | Pyruvate dehydrogenase complex E2 component | 201.2481 | 6, 25 | 13.21 | 2 |
| LACR_0691 | Pyruvate-formate lyase | 257.0184 | 14, 51 | 8.89 | 2 |
| pyk | Pyruvate kinase | 1942.121,  1618.708 | 15, 108,  17, 108 | 34.26,  37.25 | 3, 3 |
| tpiA | Triosephosphate isomerase | 8043.065,  5179.454 | 9, 67,  3, 52 | 48.02,  20.63 | 3, 3 |
|  | **Central Intermediary Metabolism** |  |  |  |  |
| metK | S-adenosylmethionine synthase | 121.3318 | 5, 18 | 14.79 | 1 |
|  | **Fatty Acid and Phospholipid Metabolism** |  |  |  |  |
| fabF | 3-oxoacyl-acyl carrier protein synthase 2 | 337.9111 | 4, 15 | 10.02 | 2 |
| fabG1 | 3-oxoacyl-acyl carrier protein reductase | 317.0391 | 4, 16 | 26.31 | 1 |
| accB | Acetyl-CoA carboxylase biotin carboxylase subunit | 149.9857 | 8, 23 | 18.68 | 1 |
| acpA | Acyl carrier protein | 20890.04,  22777.52 | 5, 42,  5, 36 | 27.4,  27.4 | 3, 3 |
| fabI | NADH-dependent enoyl-acyl carrier protein reductase | 135.2048 | 7, 26 | 22 | 1 |
|  | **Purines, Pyrimidines, Nucleosides and Nucleotides** |  |  |  |  |
| adk | Adenylate kinase | 570.4587,  860.5188 | 9, 28,  8, 22 | 38.6,  38.6 | 3, 2 |
| purA | Adenylosuccinate synthase | 124.277 | 12, 29 | 28.6 | 1 |
| carB | Carbamoyl phosphate synthase large chain | 106.5032 | 13, 30 | 4.7 | 1 |
| rmlB | dTDP-glucose-4,6-dehydratase | 102.9142 | 5, 14 | 10.57 | 1 |
| rmlA | Glucose-1-phosphate thymidylyltransferase | 185.549 | 11, 31 | 19.72 | 1 |
| guaA | GMP synthase glutamine hydrolyzing | 202.93,  171.0262 | 13, 32,  9, 36 | 15.01,  14.62 | 2, 1 |
| guaB | Inosine-5-monophosphate dehydrogenase | 435.5376,  1138.23 | 17, 52,  14, 55 | 28.8, 39.76 | 3, 3 |
| pyrE | Orotate phosphoribosyltransferase | 267.5086,  846.6959 | 4, 17,  6, 21 | 17.7,  33.49 | 1, 3 |
| deoB | Phosphopentomutase | 229.863,  318.0869 | 5, 22,  5, 26 | 11.19,  14.84 | 3, 1 |
| prsA | Ribose-phosphate pyrophosphokinase | 134.0211 | 6, 16 | 20.13 | 1 |
| prsB | Ribose-phosphate pyrophosphokinase | 102.0603 | 4, 15 | 12.15 | 1 |
| upp | Uracil phosphoribosyltransferase | 1449.865,  1466.901 | 8, 45,  7, 31 | 45.02,  39.81 | 3, 3 |
| pyrH | Uridylate kinase | 122.1289 | 4, 16 | 14.71 | 1 |
|  | **Regulatory Functions** |  |  |  |  |
| pyrR | Bifunctional protein pyrR | 760.0925,  821.9574 | 6, 28,  8, 31 | 23.12,  29.48 | 1, 1 |
| typA | GTP-binding protein TypA/BipA | 390.3837,  540.9702 | 13, 52,  13, 65 | 26.1, 23 | 2, 3 |
| codY | Transcriptional regulator | 323.343,  133.3898 | 8, 26,  4, 9 | 32.44,  29.01 | 1, 1 |
| codZ | Transcriptional regulator | 102.6482 | 3, 9 | 14.89 | 1 |
| llrC | Two-component system regulator | 1088.948,  821.0146 | 6, 29,  8, 32 | 19.74,  34.33 | 3, 3 |
|  | **Replication** |  |  |  |  |
| hslA | Hu-like DNA-binding protein | 9241.828,  7615.807 | 15, 95,  12, 65 | 68.13,  51.65 | 3, 3 |
|  | **Transcription** |  |  |  |  |
| rheA | ATP-dependent RNA helicase | 154.3606 | 9, 28 | 11.25 | 1 |
| rpoA | DNA-dependent RNA polymerase alpha subunit | 740.2064,  1014.989 | 7, 24,  7, 24 | 28.85,  22.12 | 2, 3 |
| rpoB | DNA-dependent RNA polymerase beta subunit | 165.8919 | 13, 38 | 8.36 | 1 |
| nusG | Transcription antitermination protein | 132.7495 | 7, 12 | 12.97 | 1 |
| greA | Transcription elongation factor GreA | 471.6469 | 4, 12 | 36.54 | 1 |
|  | **Translation** |  |  |  |  |
| rpsA | 30S ribosomal protein S1 | 3418.073, 2925.366 | 34, 184, 35, 188 | 52.45, 52.21 | 3, 3 |
| rpsB | 30S ribosomal protein S2 | 4652.354,  2947.827 | 11, 69,  8, 63 | 54.9,  41.96 | 3, 3 |
| rpsC | 30S ribosomal protein S3 | 2873.788,  3681.095 | 11, 62,  11, 75 | 26.73,  25.81 | 3, 3 |
| rpsD | 30S ribosomal protein S4 | 1870.133,  2167.801 | 16, 73,  15, 73 | 52.71,  53.69 | 3, 3 |
| rpsE | 30S ribosomal protein S5 | 5027.568,  5176.743 | 17, 87,  21, 95 | 54.76,  66.07 | 3, 3 |
| rpsF | 30S ribosomal protein S6 | 1390.93,  3509.92 | 9, 34,  10, 39 | 59.79,  55.67 | 3, 3 |
| rpsG | 30S ribosomal protein S7 | 3129.964,  4244.084 | 7, 45,  7, 35 | 36.13,  42.58 | 3, 3 |
| rpsH | 30S ribosomal protein S8 | 2513.234,  1304.54 | 14, 75,  8, 48 | 73.48,  54.55 | 3, 3 |
| rpsI | 30S ribosomal protein S9 | 2104.563,  4634.061 | 7, 34,  10, 43 | 30, 52.31 | 3, 3 |
| rpsJ | 30S ribosomal protein S10 | 3786.922, 8076.178 | 6, 42,  7, 46 | 39.22, 50.98 | 3, 3 |
| rpsL | 30S ribosomal protein S12 | 1277.123,  1459.528 | 8, 41,  8, 42 | 29.93,  33.58 | 3, 3 |
| rpsM | 30S ribosomal protein S13 | 1140.702,  786.1625 | 7, 22,  8, 33 | 37.19,  32.23 | 3, 3 |
| rpsN | 30S ribosomal protein S14 | 1631.731,  529.803 | 2, 13,  4, 16 | 26.23,  26.23 | 1, 1 |
| rpsP | 30S ribosomal protein S16 | 10728.4,  7999.394 | 4, 38,  6, 49 | 51.11,  47.78 | 3, 3 |
| rpsR | 30S ribosomal protein S18 | 1752.147,  1460.355 | 7, 30,  4, 21 | 35.8,  33.33 | 3, 3 |
| rpsS | 30S ribosomal protein S19 | 753.4842 | 5, 16 | 23.91 | 1 |
| rpsT | 30S ribosomal protein S20 | 4288.123,  1136.081 | 8, 35,  4, 24 | 31.17,  15.58 | 2, 2 |
| rpsU | 30S ribosomal protein S21 | 206.4021 | 3, 11 | 31.03 | 1 |
| rplA | 50S ribosomal protein L1 | 3305.582,  3039.882 | 11, 73,  15, 110 | 19.21,  26.64 | 3, 3 |
| rplB | 50S ribosomal protein L2 | 2078.184,  2224.689 | 8, 51,  10, 79 | 30.43, 35.51 | 3, 3 |
| rplC | 50S ribosomal protein L3 | 1982.263,  1773.905 | 6,30,  10, 40 | 23.19,  29.95 | 3, 3 |
| rplD | 50S ribosomal protein L4 | 1747.396,  3575.033 | 7, 43,  8, 66 | 37.02,  32.69 | 3, 3 |
| rplE | 50S ribosomal protein L5 | 2193.675,  2745.342 | 14, 74,  18, 86 | 35.56,  68.33 | 3, 3 |
| rplF | 50S ribosomal protein L6 | 6037.435,  4356.015 | 8, 53,  8, 64 | 45.51,  39.33 | 3, 3 |
| rplL | 50S ribosomal protein L7/L12 | 22413.1,  33880.27 | 17, 133,  19, 150 | 45.45,  61.16 | 3, 3 |
| rplJ | 50S ribosomal protein L10 | 2963.646,  2124.515 | 11, 59,  13, 68 | 61.4, 57.89 | 3, 3 |
| rplK | 50S ribosomal protein L11 | 3863.667,  1252.75 | 10, 58,  9, 42 | 60.28,  40.43 | 3, 3 |
| rplM | 50S ribosomal protein L13 | 1108.554,  639.0551 | 5, 22,  7, 37 | 34.46,  31.08 | 3, 3 |
| rplO | 50S ribosomal protein L15 | 2925.921,  3445.886 | 5, 34,  3, 30 | 27.21,  27.21 | 3, 3 |
| rplP | 50S ribosomal protein L16 | 480.1179,  513.6147 | 4, 21,  3, 13 | 30.66,  8.76 | 1, 3 |
| rplQ | 50S ribosomal protein L17 | 5207.507,  3722.599 | 5, 37,  6, 35 | 23.02,  37.3 | 3, 3 |
| rplR | 50S ribosomal protein L18 | 1810.697,  1269.313 | 7, 32,  8, 44 | 40.87, 45.22 | 3, 3 |
| rplS | 50S ribosomal protein L19 | 1745.723,  1415.303 | 10, 47,  7, 39 | 50.88,  42.11 | 2, 3 |
| rplT | 50S ribosomal protein L20 | 395.2145 | 4, 17 | 42.02 | 1 |
| rplU | 50S ribosomal protein L21 | 4030.36,  3285.952 | 6, 42,  9, 53 | 42.31,  45.19 | 3, 3 |
| rplV | 50S ribosomal protein L22 | 2012.804,  4320.301 | 7, 44,  9, 54 | 50.43,  34.78 | 3, 3 |
| rplW | 50S ribosomal protein L23 | 1813.187,  1608.367 | 8, 36,  5, 31 | 75.26,  40.21 | 3, 3 |
| rpmA | 50S ribosomal protein L27 | 295.9133 | 5, 20 | 25.53 | 1 |
| rpmB | 50S ribosomal protein L28 | 3159.694,  3189.104 | 9, 48,  8, 45 | 51.56,  62.5 | 3, 3 |
| rpmC | 50S ribosomal protein L29 | 4504.277,  4382.438 | 7, 44,  6, 29 | 39.13,  42.03 | 3, 3 |
| rpmD | 50S ribosomal protein L30 | 15892.67,  9235.415 | 18, 124,  15, 89 | 69.49,  76.27 | 3, 3 |
| rpmE2 | 50S ribosomal protein L31 type B | 8746.157,  8541.646 | 7, 54,  5, 33 | 44.44,  62.96 | 3, 3 |
| rpmF | 50S ribosomal protein L32 | 8620.994,  6736.098 | 1, 10,  1, 11 | 27.59,  28.07 | 1, 3 |
| rpmG1 | 50S ribosomal protein L33 1 | 1533.972 | 7, 34 | 57.14 | 1 |
| rpmJ | 50S ribosomal protein L36 | 2205.412,  1336.766 | 1, 10,  2, 14 | 26.32,  60.53 | 1, 2 |
| pepC | Aminopeptidase C | 166.7645 | 11, 26 | 22.71 | 2 |
| pepN | Aminopeptidase N | 239.39,  332.6772 | 14, 35,  4, 13 | 9.34,  5.08 | 1, 1 |
| argS | Arginyl-tRNA synthetase | 287.3493,  420.1981 | 6, 21,  12, 39 | 10.46,  29.61 | 2, 1 |
| pepV | Dipeptidase | 379.2519 | 5, 13 | 13.14 | 1 |
| fusA | Elongation factor G | 1314.489,  1923.685 | 32, 155,  29, 158 | 41.89, 38.65 | 3, 3 |
| efp | Elongation factor P | 137.6273 | 7, 25 | 14.59 | 1 |
| tsf | Elongation factor Ts | 3630.041,  2860.844 | 15, 96,  13, 89 | 39.47,  38.6 | 3, 3 |
| tuf | Elongation factor Tu | 18900.78,  19021.71 | 31, 288,  35, 284 | 42.03,  49.62 | 3, 3 |
| ileS | Isoleucyl-tRNA synthetase | 360.9346 | 9, 23 | 7.83 | 1 |
| pepT | Peptidase T | 174.2384 | 7, 20 | 8.96 | 2 |
| ppiB | Peptidyl-prolyl cis-trans isomerase | 415.9875,  400.4594 | 5, 19,  5, 22 | 33.16,  23.47 | 3, 2 |
| pheT | Phenylalanyl-tRNA synthetase beta chain | 111.4441 | 8, 15 | 27.4 | 1 |
| pepQ | Proline dipeptidase | 355.9358,  219.2688 | 5, 17,  9, 36 | 19.89,  29.83 | 2, 2 |
| pepO | Prolidase | 156.89 | 9, 32 | 18.51 | 1 |
| frr | Ribosome recycling factor | 1257.472,  1868.146 | 6, 25,  8, 37 | 38.38,  48.65 | 3, 3 |
| serS | Seryl-tRNA synthetase | 201.3293,  101.6891 | 10, 24,  5, 16 | 15.84,  12.53 | 1, 1 |
| thrS | Threonyl-tRNA synthetase | 152.6513 | 6, 21 | 10.53 | 1 |
| infA | Translation initiation factor IF-1 | 385.7959 | 1, 11 | 16.67 | 1 |
| tyrS | Tyrosyl-tRNA synthetase | 131.0451 | 13, 27 | 11.46 | 1 |
| LACR_1813 | Xaa-Pro aminopeptidase | 199.5197 | 9, 30 | 25.41 | 1 |
|  | **Transport and Binding Proteins** |  |  |  |  |
| ptsl | Phosphoenolpyruvate protein phosphotransferase | 492.7552,  210.1151 | 11, 53,  13, 58 | 21.91,  29.74 | 3, 3 |
| malE | Maltose ABC transporter substrate binding protein | 281.6267 | 13, 36 | 34.47 | 1 |
|  | **Other Categories** |  |  |  |  |
| clpB | ClpB protein | 156.552 | 19, 46 | 10.5 | 2 |
| cspE | Cold shock protein E | 6208.647,  4910.865 | 7, 49,  5, 35 | 38.46,  38.46 | 3, 3 |
| grpE | Stress response protein E | 298.0949 | 3, 9 | 17.32 | 1 |
|  | **Hypothetical Proteins** |  |  |  |  |
| yhjA | General stress protein, CsbD superfamily | 3828.877 | 6, 34 | 39.24 | 3 |
| ytjD | Nitroreductase family protein | 215.2552 | 4, 17 | 13.37 | 1 |
| llmg_1773 | Putative uncharacterized protein | 5686.698,  3828.877 | 7, 47,  6, 34 | 39.24,  39.24 | 3, 3 |
| llmg_2049 | Putative uncharacterized protein | 278.6473 | 3, 14 | 31.58 | 1 |
| SA8A11-2 | SA8A11-2 protein | 281.7025 | 4, 15 | 15.69 | 1 |
| LACR_1462 | UDP-glucose pyrophosphorylase | 254.0043 | 2, 10 | 7.67 | 2 |

*The PLGS score, the number of peptides and the protein coverage correspond to the best LC-MS/MS acquisition among triplicates. **a** indicates the proteome identified in the mid-exponential phase and **b** indicates the proteome identified in the early stationary phase. For the number of peptides, **a/b^1^** and **a/b^2^** indicate the number of matched peptides per protein and the number of matched fragments per protein respectively. The protein identification was manually validated for proteins with PLGS score below 200.
